# Mitigating Plasma Etch-Induced Negative Charge Trapping in 2.7 kV β‑Ga_2_O_3_ (001) Trench Schottky Barrier Diodes Using H_3_PO_4_ Treatment

**DOI:** 10.1021/acsaelm.5c02537

**Published:** 2026-03-05

**Authors:** Min-Yeong Kim, Aditya Kundapura Bhat, Sai Charan Vanjari, Matthew D. Smith, Martin Kuball

**Affiliations:** † Center for Device Thermography and Reliability (CDTR), 1980 University of Bristol, Bristol BS8 1TL, U.K.; ‡ Innovation and Knowledge Centre REWIRE, 1980 University of Bristol, Bristol BS8 1TL, U.K.

**Keywords:** Ga_2_O_3_, trench Schottky barrier diodes, interface traps, H_3_PO_4_ treatment, etch damage, bias stress instability, high temperature

## Abstract

Stable β-Ga_2_O_3_ (001) trench Schottky barrier diodes (TSBDs) with a Baliga’s figure-of-merit (BFOM) of 0.7 GW cm^–2^ were demonstrated by reducing the Al_2_O_3_/Ga_2_O_3_ interface state trap density using a H_3_PO_4_ surface treatment during device fabrication. TSBDs with fins oriented along different directions have been studied, wherein devices with [010] fin orientation exhibited a low specific on-resistance (*R*_on,sp_) of 11 mΩ cm^2^ and a breakdown voltage (*V*_br_) of up to 2.7 kV with H_3_PO_4_ treatment. Reliability testing using sequential voltage stress up to a reverse bias of −1.2 kV showed a degradation in *R*_on,sp_ by 20% in untreated devices but only by 9% in those with the H_3_PO_4_ surface treatment. TCAD simulations confirm that the H_3_PO_4_ treatment mitigates the density of negative interface charges, highlighting the effectiveness of the acid treatment in controlling defect-mediated instabilities. Furthermore, high-temperature bias stress tests demonstrated that [010]-oriented TSBDs achieved superior thermal and electrical stability after the treatment, eliminating the 10% *R*_on,sp_ increase observed in untreated devices. These results establish H_3_PO_4_ surface treatment as an effective strategy for enhancing the robustness of β-Ga_2_O_3_ power devices under combined thermal and electrical stress.

## Graphical abstract

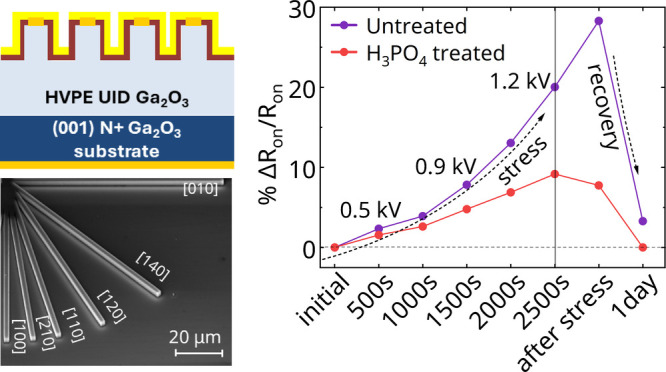

## Introduction

Beta-gallium oxide (β-Ga_2_O_3_) has attracted considerable attention for its ability to enable compact and energy-efficient power electronic devices, addressing the increasing power requirements of transportation, computing, and data center applications.^1–3^ Its ultrawide bandgap (4.9 eV) results in a high critical electric field (8 MV cm^–1^) and an associated high Baliga’s figure-of-merit (BFOM).^4–9^ Furthermore, the availability of potentially low-cost single-crystal substrates has positive implications for commercial uptake in mass-market power electronics applications. Recent demonstrations in vertical unipolar Ga_2_O_3_ devices include vertical trench Schottky barrier diodes (TSBDs) of up to 4 kV^10^ and transistors reaching over 10 kV.^11^ Surface field engineering in TSBDs, i.e., reduced surface field (RESURF) designs, is essential to fully exploit the high critical field strength of Ga_2_O_3._ ^12^ Due to the lack of p-type doping in Ga_2_O_3_, implementation of conventional RESURF structures though is limited.^6,12,13^ TSBDs employ trench structures that exploit sidewall metal-oxide-semiconductor (MOS) capacitance to redistribute electric fields, offering a RESURF-like benefit in device performance.^5,6,14^ However, they introduce MOS interfaces along dry-etched sidewalls, where interface states and etch-induced deep-level defects can degrade turn-on voltage, on-resistance, current collapse, and reliability.^15–17^ Various approaches have been extensively investigated to eliminate interface states that degrade device operation. Recent studies have reported that vacuum annealing can restore the on-current to levels comparable to pre-etch devices,^18^ while atomic nitrogen treatment has been shown to improve breakdown voltage (*V*_br_).^19^ However, these efforts have focused exclusively on static characteristics at room temperature (RT), without considering device stability under bias stress or high-temperature operating conditions. Furthermore, due to the anisotropic nature of Ga_2_O_3_, the orientation of the sidewall relative to the crystallographic axes impacts interface state formation. Previous studies have reported that TSBDs with fins aligned along the [010] direction exhibit a relatively low specific on-resistance (*R*_on,sp_), which has been attributed to the favorable crystallographic orientation of the fin sidewalls.^4,20^ Surface preparation and treatments, such as chemical treatment or postdeposition annealing (PDA), have been used to improve interface quality.^21–24^ These studies have primarily focused on surfaces that were not dry-etched and have not considered the orientation of gallium oxide. Few studies have attempted to reduce the effect of interface states via chemical treatment, as most acids, such as HCl, H_3_PO_4_, HF and HNO_3_, are typically used as wet etching solutions.^25–27^ Initial reliability studies on Ga_2_O_3_ SBD showed reasonable reliability,^10,28^ though still some degradation was observed, which needs to be addressed if this technology is to be implemented in real-world applications. In the present work, we investigated the electrical characteristics and reliability of Ga_2_O_3_ TSBDs through a comprehensive analysis, where H_3_PO_4_ treatment is employed to mitigate etch-induced charge trapping at the MOS interface and to assess its impact on device performance and stability. We first examine the dependence of fin orientation on device stability under repeated low positive-bias stress at RT. Using the most stable fin-oriented TSBDs, we then examined device stability under negative-bias stress of up to −1.2 kV. The impact of negative interface charge density on device performance was further analyzed using simulations, which were compared with the experimental results. Finally, the most stable TSBDs are subjected to repeated current measurements at 150 °C to assess their reliability under harsh thermal conditions.

## Experimental Section

Figure 1 shows the fabrication process flow and cross-section of the vertical TSBDs. The devices were fabricated on a (001)-oriented β-Ga_2_O_3_ wafer grown from the melt by edge-defined film-fed growth (EFG) with hydride vapor phase epitaxy (HVPE)-grown epitaxial layers from Novel Crystal Technology, Inc. The epitaxial structure consisted of an unintentionally doped 11 μm thick Ga_2_O_3_ layer (carrier concentration *n* ≈ 1.3 × 10^16^ cm^–3^) on a Sn-doped (001) Ga_2_O_3_ substrate (*n* ≈ 5.4 × 10^18^ cm^–3^, 640 μm thick). After cleaning with acetone, isopropanol, and deionized water, the wafer was treated with piranha solution (1:3 H_2_O_2_/H_2_SO_4_) to remove surface contaminations. The fins were defined using electron-beam lithography (EBL) with a width and spacing of 1.5 μm, using a SiO_2_ (150 nm) and Ni (150 nm) hard mask, patterned by inductively coupled plasma reactive ion etching (ICP-RIE) with a BCl_3_/Ar mixture (aligned to crystallographic orientations, as shown in Figure 1) and with an etch depth of 1.4 μm. The Ni mask was then removed by a piranha wet etch process. Samples were subsequently treated with H_3_PO_4_ solution (>85% concentration) for 0 or 10 min at RT without agitation, denoted as untreated and H_3_PO_4_ treated, respectively. The protective SiO_2_ layer on the top fin surface was then removed by a buffered oxide etch (BOE). A 160 nm aluminum oxide (Al_2_O_3_) dielectric layer was subsequently deposited by plasma-assisted atomic layer deposition using a trimethylaluminum precursor and oxygen plasma and annealed at 500 °C for 10 min in O_2_. Contact openings were patterned by EBL, and Al_2_O_3_ was etched using an ICP-RIE process. Ni/Cr (20/200 nm) was deposited by angled sputtering as the Schottky contact on the top side of the sample, while Ti/Au (20/200 nm) was sputtered as the blanket cathode contact on the back side. Electrical measurements, including off-state stress, were performed using a Keithley 2657A high-voltage source in conjunction with a Keithley 2636B system.

**Figure 1:**
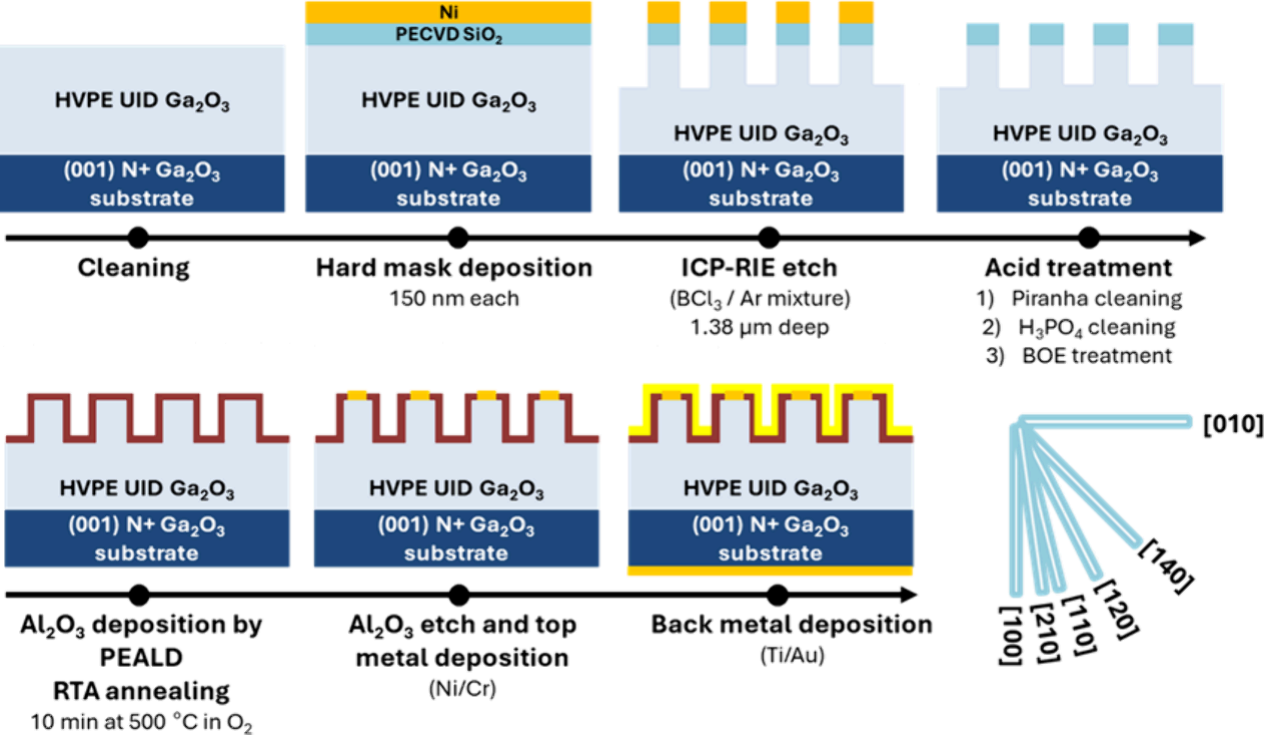
Schematics of the trench Schottky barrier diode (TSBD) with fabrication process flow and different fin orientations implemented in this study.

## Results and Discussion

Figure 2a presents the repeated forward current density–voltage (*J–V*) characteristics of the untreated TSBDs under voltage sweeps from 0 to 5 V. The results reveal strong fin-orientation-dependent forward *J–V* characteristics. Compared to the initial sweep, subsequent measurements exhibit a positive shift in turn-on voltage (*V*_on_) of approximately +1.0 and +1.5 V for most orientations. Here, *V*_on_ is defined at a current density of 1 μA cm^–2^. Notably, [010]-oriented TSBDs maintain a stable *V*_on_ near 0.5 V even after repeated measurements, indicating good forward-bias stress stability at RT. Such positive *V*_on_ shifts are commonly associated with electron trapping at the semiconductor/dielectric interface.^29,30^ In contrast, Figure 2b shows that H_3_PO_4_-treated TSBDs exhibit significantly reduced *V*_on_ shifts for [140]- and [120]-oriented TSBDs, after 10 min of surface treatment, while [010]-oriented TSBDs remain unchanged, preserving the highest on/off ratio and a stable *V*_on_ of 0.6 V. Other orientations of TSBDs ([110], [210], and [100]) still displayed noticeable shifts, even after H_3_PO_4_ treatment, albeit smaller than in untreated TSBDs. Figure 2c summarizes the initial turn-on voltage (*V*_on,initial_) values across different fin orientations, revealing that [010]-oriented TSBDs consistently achieve the lowest *V*_on,initial_. After H_3_PO_4_ treatment, the variation in *V*_on,initial_ among other orientations becomes less pronounced, suggesting improved interface quality. To quantify forward-bias stress effects, Figure 2d plots the median Δ*V*_on_, which is differential between the fourth and initial measurements. For [120]- and [140]-oriented TSBDs, Δ*V*_on_ decreases significantly from ∼1.4 to ∼0.3 V after treatment, whereas [010] remains almost unaffected under low-bias stress conditions at RT. Orientation-dependent variations in electrical behavior have been attributed to differences in interface and border trap densities, which govern charge-trapping dynamics.^20,31,32^ The electrical characteristics suggest that H_3_PO_4_ treatment can suppress the effects of surface defects in the TSBDs, potentially mitigating interface states at the Al_2_O_3_/Ga_2_O_3_ interface. This finding underscores the critical role of crystallographic orientation and surface treatment in enhancing the devices’ forward-voltage-stress reliability.

**Figure 2:**
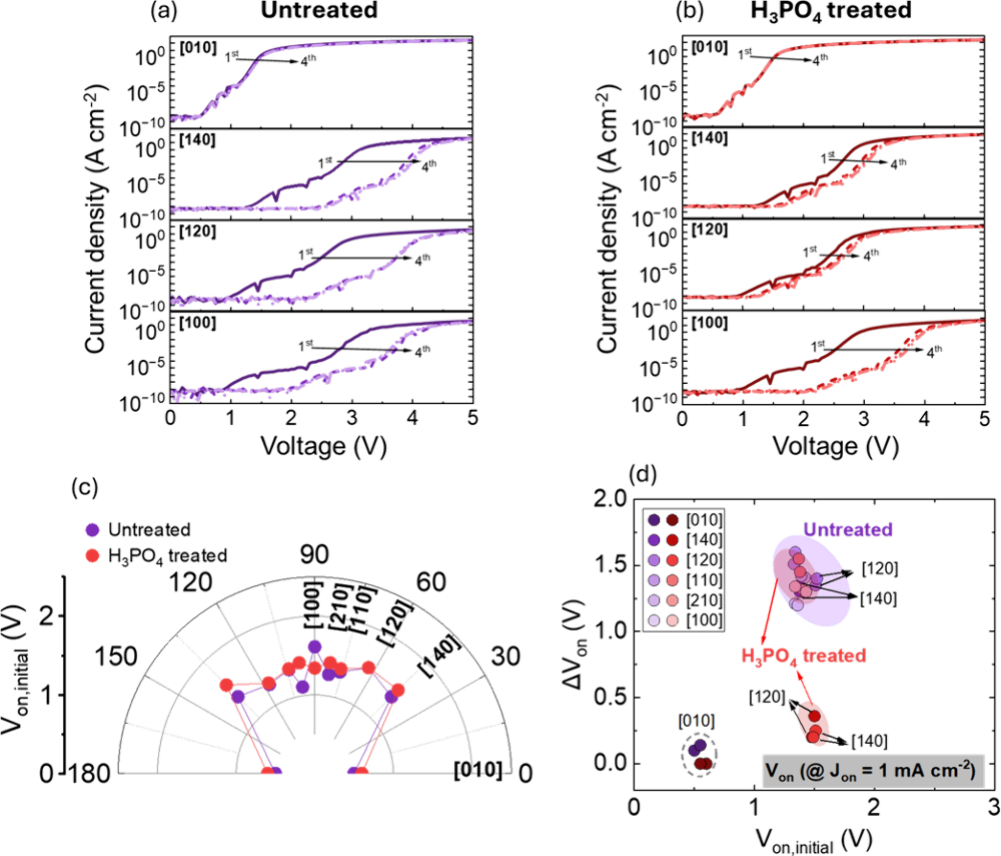
Repeated current density–voltage (*J–V*) characteristics from 0 to 5 V of Ga_2_O_3_ TSBDs with different fin orientations: (a) untreated and (b) H_3_PO_4_-treated TSBD. (c) *V*_on,initial_ as a function of fin orientation, untreated and H_3_PO_4_ treated. (d) Summary plots of Δ*V*_on_ vs *V*_on,initial_, measured at RT under repeated voltage sweeps from 0 to 5 V. Δ*V*_on_ denotes the shift in *V*_on_ between the fourth and the initial measurements.

Figure 3a shows the linear-scale forward *J–V* characteristics along with the corresponding *R*_on,sp_ plots for the best devices from both samples, i.e., TSBDs with [010] fin-orientation, which exhibited the lowest *V*_on_ under repeated forward voltage sweeps. After H_3_PO_4_ treatment, the on-current increased noticeably, resulting in a reduction of *R*_on,sp_ (defined at a forward bias of 5 V) from approximately 13 ± 0.02 mΩ cm^2^ for untreated devices to 11 ± 0.01 mΩ cm^2^ for H_3_PO_4_-treated devices. Device-to-device variation was compared across the five devices for each condition. This trend was consistently observed across all measured devices, with 10 devices evaluated for each sample. Off-state characteristics were also evaluated for multiple devices with different fin orientations, and representative results are shown in Figure 3b. The values of *V*_br_ exhibited minimal dependence on orientation, consistent with previous reports,^20^ but H_3_PO_4_ treatment produced a slight improvement. The median *V*_br_ increased from 2.5 kV for untreated TSBDs to 2.7 kV after H_3_PO_4_ treatment, accompanied by reduced variability among devices. The correlation between *R*_on,sp_ and *V*_br_ is summarized in Figure 3c, where [010]-oriented TSBDs, featuring fin sidewalls formed by (100) and ( 1®00) crystallographic planes, consistently demonstrate the lowest *R*_on,sp_, while H_3_PO_4_-treated samples achieve higher *V*_br_. This highlights the significant impact of the H_3_PO_4_ treatment on the BCl_3_ ICP-RIE etched trench sidewall surface that is formed during the fabrication process by ICP-RIE etch. H_3_PO_4_ treatment was performed at RT to minimize etch rates and prevent significant changes in fin dimensions.^33,34^ Cross-sectional scanning electron microscopy (SEM) confirmed that untreated and H_3_PO_4_-treated TSBDs exhibit nearly identical fin geometries, with widths of ∼1.5 μm and depths of ∼1.4 μm, as shown in Figure 3d,e. Despite no significant change in the physical fin structure, the electrical performance showed a clear dependence on H_3_PO_4_ treatment. This aligns with existing reports concluding that the on-resistance of TSBDs is influenced not only by the fin geometry but also by charge trapping at the dielectric/semiconductor interface.^10,35,36^ As a result, the BFOM in [010]-oriented TSBD with H_3_PO_4_ treated reaches 0.7 GW cm^–2^, which is a 45% improvement.

**Figure 3:**
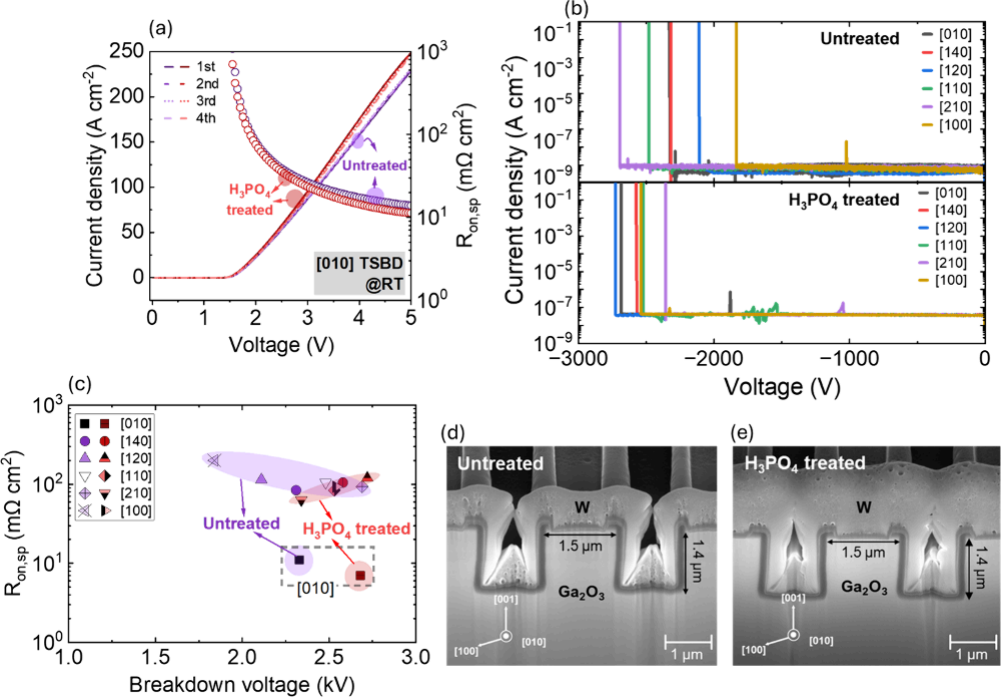
(a) Representative *J–V* characteristics and *R*_on,sp_ of a representative untreated and H_3_PO_4_-treated [010]-oriented TSBDs. (b) Breakdown characteristics of Ga_2_O_3_ TSBDs with different fin orientations depending on H_3_PO_4_ treatment. (c) *R*_on,sp_ vs breakdown voltage for untreated and H_3_PO_4_-treated samples with different fin orientation. Scanning electron microscopy (SEM) images of Ga_2_O_3_ TSBDs with [010]-oriented fins for (d) untreated and (e) H_3_PO_4_ treated, recorded at a tilt angle of 52° in the SEM with respect to the sample surface; no significant change in fin width is observed after H_3_PO_4_ treatment. The fin dimensions are approximately 1.5 μm in width and 1.4 μm in depth under both conditions. The tungsten layer in panels (d,e) was deposited for surface protection during the FIB cross-sectioning of the device structure.

We evaluated device stability using repeated *J–V* measurements following negative-bias stress, performed on untreated and H_3_PO_4_-treated [010]-oriented TSBDs. The current was measured before (initial) and after applying a negative-bias stress voltage for 500 s with a ramp rate of −4 V s^–1^, followed by remeasurement of the forward *J–V* characteristics and subsequent repeating of the stress at increasing reverse biases. On-current reduces with increasing bias stress, as shown in Figure 4a. A larger current degradation was observed for untreated devices compared to that of H_3_PO_4_-treated TSBDs. The plot of %Δ*R*_on_/*R*_on_ vs stress and recovery time in Figure 4b shows a clear trend of increasing specific on-resistance (*R*_on,sp_) under negative-bias stress, followed by partial recovery during the relaxation period. After applying a maximum stress bias of −1.2 kV, the *R*_on,sp_ (defined at a voltage of 5 V) of untreated TSBD increased by 20% relative to its initial value, while H_3_PO_4_-treated TSBD showed only a 9% increase. Recovery was evaluated by measuring the forward *J–V* characteristics after the final stress. Untreated TSBDs exhibited incomplete recovery after 1 h, with 15% increase in *R*_on,sp_ compared to the initial state. In contrast, the H_3_PO_4_-treated TSBDs exhibited only a 3.8% increase with respect to the value measured prior to stress after 1 h, indicating near-complete recovery.

**Figure 4:**
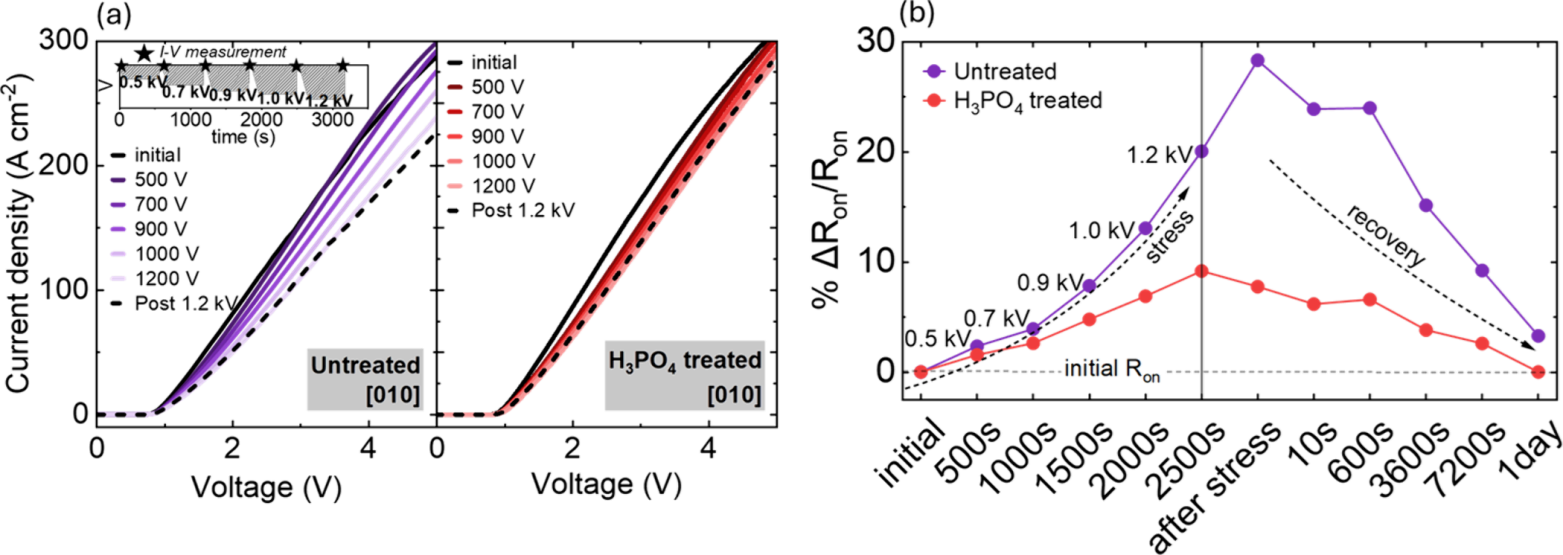
(a) Forward *J–V* characteristics of Ga_2_O_3_ TSBDs of untreated and H_3_PO_4_-treated samples after 500 s stresses at various reverse voltages. Inset: schematics of stress–measurement sequence for negative-bias stress. (b) Percentage of *R*_on_ change as a function of stress and rest time.

The effect of bias stress on charge trapping can be emulated through the inclusion of fixed charges applied to TSBD device simulations in Silvaco ATLAS. To ensure realistic simulation results, a fin corner curvature of 6.37 × 10^4^ m^–1^, extracted from the cross-section SEM images, was incorporated into the structure. Changes in *J–V* characteristics resulting from the inclusion of negative charges on the sidewall and bottom-corner regions of the fin are compared, as shown in Figure 5a. When charging occurs along the fin sidewalls and corners, the *J–V* curve exhibits both a positive *V*_on_ shift and increased *R*_on,sp_. Such a trend was observed experimentally with repeated measurements for all fin orientations except the [010]-orientation (Figure 2); this highlights the impact of interface trapping on the turn-on properties of TSBDs. When a positive bias is applied to the anode, electrons within the Ga_2_O_3_ layer accumulate near the Ga_2_O_3_/Al_2_O_3_ interface, as Al_2_O_3_ is an insulating layer and does not allow for electron penetration. These electrons get trapped at pre-existing interface defects. Consequently, subsequent forward sweeps require a higher positive voltage to counter the effect of the trapped charges, manifesting as a positive *V*_on_ shift. In contrast, when charging is concentrated at the fin corners, as shown in Figure 5b, the *J–V* curve primarily exhibits increased *R*_on,sp_ without a significant *V*_on_ shift, as seen experimentally under high reverse bias stress at −1.2 kV in Figure 4a. Interestingly, the [010]-oriented TSBDs, which exhibit superior stability under low forward-voltage conditions, become vulnerable to trapping only under high negative-bias stress. Although [010]-oriented TSBDs inherently apparently possess fewer interface traps than other orientations,^20^ high reverse-bias conditions induce interface charging effects. Electric-field crowding at the bottom corner of the fin far exceeds the field intensity along the sidewalls.^10,37^ Interface states are present along both the fin sidewalls and the corners. However, the potential difference between the sidewalls and the fin center is small, leading to a smaller electric field that limits the carrier exchange between the anode and these interface states. As a result, most interface states located near the fin corners should effectively be charged. The corresponding energy band diagram under these conditions illustrates the enhanced potential for electron trapping in these localized regions, as shown in Figure 5c,d.

**Figure 5:**
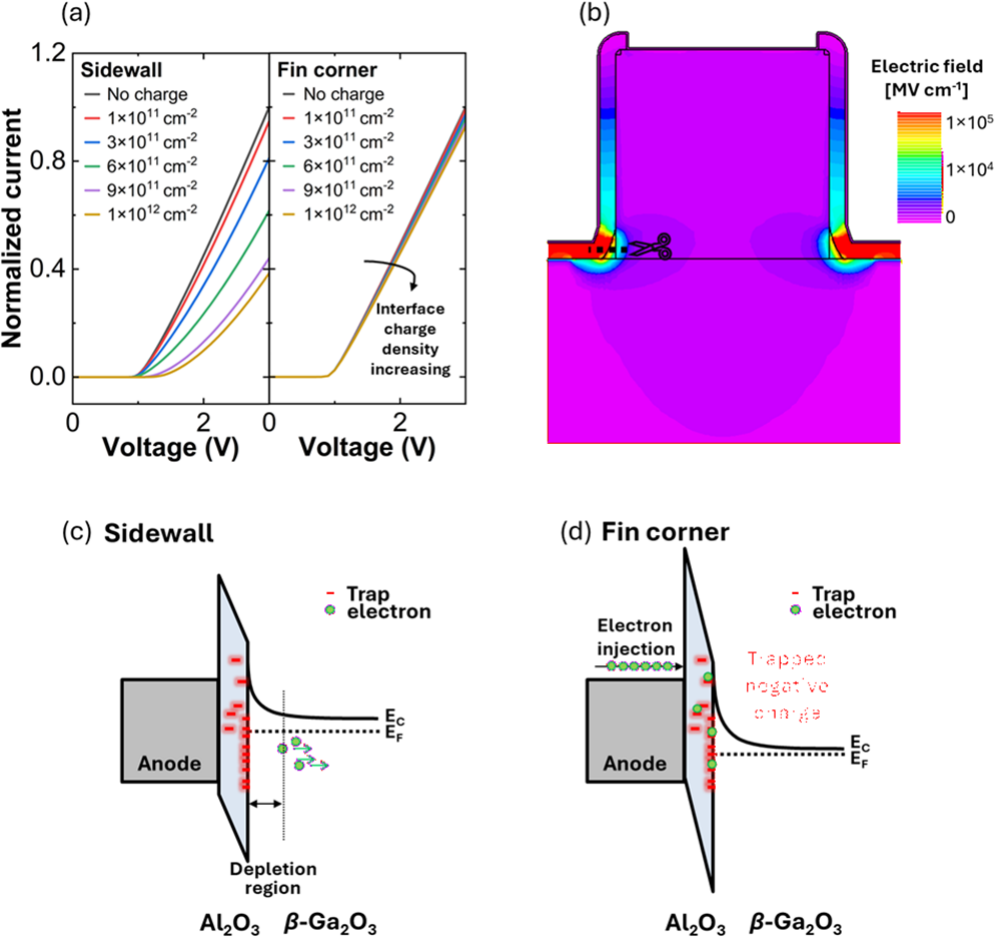
(a) Simulated current–voltage characteristics of TSBDs with varying interface charge densities at the sidewall and bottom corner of the fins. The interface charge at the bottom of corner was applied within a 0.2 μm region of the corner along both the *x*-and *y*-axes. (b) Simulated electric field distribution at −1.2 kV. Schematic energy band diagram at the (c) sidewall and (d) bottom corner of the fin.

To further explore these findings, trap density was fitted in the TCAD simulation to fit the experimental normalized *R*_on,sp_ values for [010]-oriented TSBDs, from untreated and H_3_PO_4_-treated samples, as shown in Figure 6. *R*_on,sp_ increases with both stress voltage and interface charge density in both devices as a function of electric field, although the change is less pronounced in H_3_PO_4_-treated TSBDs. Untreated TSBDs show good agreement with simulations assuming an initial interface charge density at the corner of 3 × 10^11^ cm^–2^, while H_3_PO_4_-treated TSBDs correspond to an initial density of less than 9 × 10^10^ cm^–2^. These results confirm our earlier conclusion that H_3_PO_4_ treatment can effectively reduce negative interface charges or the border oxide trap at the [010] fin corners, mitigating current degradation.

**Figure 6:**
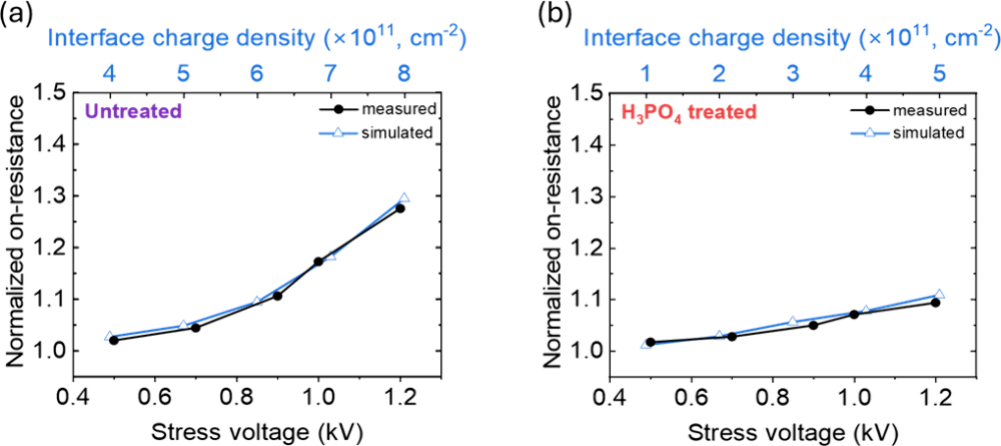
Simulated normalized *R*_on,sp_, compared to the measurement, as a function of stress voltage for devices (a) untreated and (b) H_3_PO_4_ treated by adjusting the interface charge density at fin corners to fit the experimental data.

Having established the most stable operation for the [010]-oriented TSBDs at RT, we also examined their reliability at high temperature (150 °C). Figure 7a presents the forward-bias characteristics measured at 150 °C, compared to results obtained at RT. Both untreated and H_3_PO_4_-treated devices exhibited a reduction in on-current at higher temperatures, which is expected considering the enhanced electron scattering at higher temperatures.^38,39^ The most notable observation concerns the behavior under repeated forward sweep measurements at a high temperature. Similar to RT testing, H_3_PO_4_-treated TSBDs maintain stable performance across multiple bias cycles at 150 °C. In contrast, untreated devices showed a progressive decrease in on-current with each successive measurement, accompanied by *V*_on_ shift of approximately +0.2 V. A detailed comparison in Figure 7b reveals that while H_3_PO_4_-treated devices exhibit negligible variation between measurements, untreated devices experience a 10% increase in *R*_on,sp_ after the fourth cycle relative to the initial measurement. This observed degradation aligns with the previously proposed mechanism: repeated bias stress promotes electron trapping at interface states, resulting in a negative charge accumulation. In particular, we note that untreated [010]-oriented TSBDs exhibit an increase in *R*_on,sp_ at high temperatures, indicating that interface states that have a negligible influence on electrical characteristics under low-voltage stress at RT become thermally activated and accessible for electron trapping at high temperatures, where sufficient thermal energy enables electron trapping and enables negative charging. Such activation of deep-level traps can significantly affect the device parameter, including *V*_on_, and *R*_on,sp_. For β-Ga_2_O_3_ devices, deep-level traps are reported to become active within the temperature range of 146–172 °C,^40–42^ which can contribute to the observed instability at elevated temperature. Our results indicate that both traps active at RT and high temperatures can be effectively mitigated through H_3_PO_4_ treatment. Because the traps require activation energy, their activation occurs at elevated temperatures under combined thermal and electrical stress. As a result, untreated TSBDs have markedly higher instability. Therefore, the impact of H_3_PO_4_ treatment on improving stability is even more critical under these harsher conditions to enable robust and reliable device operation.

**Figure 7:**
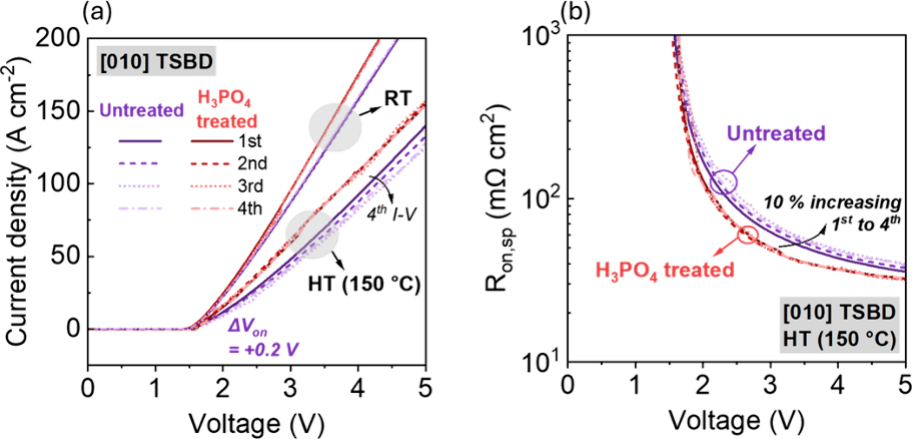
(a) Repeated *J–V* characteristics from 0 to 5 V of Ga_2_O_3_ [010]-oriented TSBDs with [010] orientation measured at RT and high temperature (150 °C), with each device measured through successive forward sweeps. (b) Extracted *R*_on,sp_ for untreated and H_3_PO_4_-treated [010]-oriented TSBDs.

## Conclusions

We studied the effect of H_3_PO_4_ surface treatment on the electrical performance and reliability under high-temperature and bias stressing in β-Ga_2_O_3_ TSBDs using measurements and simulations. At RT, the treatment ensured stable operation for [140]- and [120]-oriented TSBDs, while the [010]-oriented TSBD achieves a notable performance increase, with *R*_on,sp_ reduced from 13 to 11 mΩ cm^2^ and *V*_br_ increased from 2.5 to 2.7 kV, resulting in a BFOM of approximately 0.7 GW cm^–2^. Under sequential voltage stress at reverse bias of up to −1.2 kV, untreated devices exhibited a 26% increase in *R*_on,sp_, compared to only 11% in H_3_PO_4_-treated devices, and only 1 h was required for near-complete recovery. Furthermore, high-temperature reliability was confirmed at 150 °C, where H_3_PO_4_-treated TSBDs retained stable performance across repeated measurements, in contrast to untreated devices that exhibited a 10% *R*_on,sp_ increase and a positive *V*_on_ shift. Overall, these findings underscore the importance of controlling interface charges and surface defects through surface treatments during device processing for enabling robust and reliable β-Ga_2_O_3_ TSBD operation under combined electrical and thermal stress, paving the way for high-voltage power electronics in harsh environments.
